# “Natural” fibers in lakes: A 150-year sedimentary perspective on persistence

**DOI:** 10.1016/j.isci.2026.114904

**Published:** 2026-02-03

**Authors:** Thomas Stanton, Antonia Law, Carry Somers, Savannah Worne, Kelly J. Sheridan, Chimdia Kechi-Okafor, Alana Wheat, Alexander Wood, Anna Bateman, Naomi Richardson, Edwin Baynes, David B. Ryves, Pawel Gaca, Andrew Cundy, Deirdre McKay

**Affiliations:** 1 Department of Geography and Environment, Loughborough University, Loughborough LE11 3TU, UK; 2 School of Geography, University of Nottingham, Nottingham NG7 2RD, UK; 3 Fashion Revolution, London W1W 8DA, UK; 4 League of Artisans, Leek, Staffordshire ST13 5AJ, UK; 5 Centre for Forensic Science, Department of Applied Sciences, Faculty of Health and Life Sciences, Northumbria University, Newcastle Upon Tyne NE1 8ST, UK; 6 Department of Geography and Environmental Sciences, Faculty of Engineering and Environment, Northumbria University, Newcastle Upon Tyne NE1 8ST, UK; 7 School of Life Sciences, Keele University, Newcastle-under-Lyme ST5 5BG, UK; 8 Independent Scholar, Loughborough, UK; 9 GAU-Radioanalytical, School of Ocean and Earth Science, National Oceanography Centre (Southampton), University of Southampton, Southampton SO14 3ZH, UK

**Keywords:** Environment, Pollution, Environmental assessment

## Abstract

Natural fiber textiles, such as cotton, are widely marketed as greener, biodegradable materials within the fashion and textiles industry. However, contemporary environmental assessments of whole (plastic and non-plastic) textile fiber pollution regularly find natural, not plastic, fibers dominate environmental samples. Here we combine palaeolimnological, archival, and forensic science methodologies to evaluate long-term fiber preservation in sediments. We recover individual textile fibers from a unique 150-year lake sediment record from Rudyard lake, Staffordshire, UK. Between c.1876 and c.1979, all bar two fibers recovered from this sediment were natural. After c.1979, fiber accumulation rates increase, driven primarily by an increase in cotton accumulation. These data challenge assumptions of natural fiber biodegradability and add to the technofossil record of historic human activity. We conclude that there is a pressing need to reconsider whether natural textile fibers are as benign as is largely assumed, particularly in pursuit of plastic alternatives for fashion and textiles.

## Highlights

• Natural textile fibers persist in lake sediment records, alongside plastic fibers
• Only two plastic fibers were recovered from sediments predating 1979
• Natural textile fibers are potential technofossil markers for human activity
• Biodegradation of natural fibers cannot be assumed

## Graphical abstract

**Figure:**
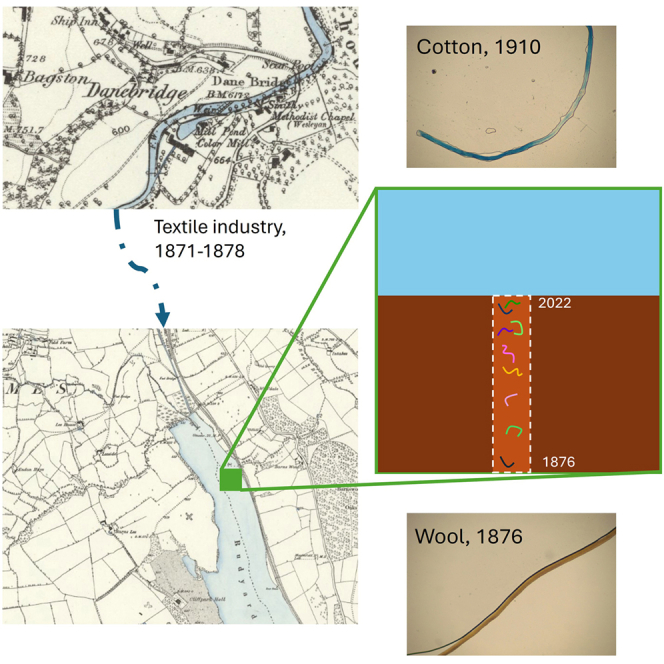

## Introduction

Where research into contemporary environmental pollution has considered entire textile fiber populations, non-plastic textile fibers (e.g., cotton and wool) are consistently identified as the dominant fiber type,^1^^,^^2^^,^^3^^,^^4^ particularly cellulosic fibers.^5^ While this knowledge is relatively new to the field of environmental pollution research, it is established in complementary disciplines. Forensic science research has long-reported natural fiber dominance in textile fiber populations.^6^^,^^7^^,^^8^^,^^9^ Within the archaeological sciences there is also extensive evidence of long-term (centuries to millennia) preservation of macroscopic textile remains^10^^,^^11^ and even of individual textile fibers of plant and animal origin.^12^

However, environmental pollution research has, to date, focused more effort on characterizing plastic textile fibers, such as polyester, acrylic, and polyamide, which are pervasive anthropogenic particles that frequently dominate environmental microplastic samples.^13^ As part of a body of research focusing on plastic pollution, studies of these petrochemical-derived fibers have associated them with environmental harms including ingestion,^14^^,^^15^ chemical leaching^16^ and chemical transport.^17^ This has led to public promotion of natural fibers over plastic fibers in material published by leading environmental advocacy organizations.^18^^,^^19^^,^^20^ Despite the best scientific evidence demonstrating that natural textile fibers outnumber plastic fibers in the environment today, and that they will not all biodegrade, the predominance, persistence and impacts of natural fibers remains a critical environmental knowledge gap. A lack of knowledge about the fate of natural fibers in the environment is of considerable consequence to sustainability claims within the fashion and textiles industry, and of related policy and legislation that evidence from environmental research informs. Accurately assessing textile fiber pollution therefore requires integration of approaches from complementary disciplines.^21^

### What are non-plastic and “natural” fibers?

Non-plastic fibers fall into three broad categories: plant derived (e.g., cotton, flax, hemp), animal derived (e.g., wool, cashmere, silk), and plant regenerated (e.g., viscose, lyocell). Regenerated textile fibers undergo considerable modification from their natural material sources but are “natural” in their origin. Here we focus on natural fibers—those derived from plant and animal sources. The processing of natural fibers for textile applications involves extensive use of chemicals to clean fibers, altering their chemistry and polymeric structure (e.g., mercerizing of cotton), and providing them with desirable aesthetic and technical characteristics (e.g., color, odor control, durability, and antibacterial properties) (*23*, *24*). People have been processing natural fibers for textiles for millennia.^22^ The increasing sophistication of anthropogenic manipulations to natural fibers, by which they are often fundamentally altered, therefore renders them inherently *unnatural*. Though these fibers are inherently unnatural, we use the term ’natural fibers’ here in line with fashion and textiles industry-standard terminology.

### Natural fiber biodegradation

Natural textile fibers are widely assumed to rapidly biodegrade in the environment. Hence, natural fibers are often excluded from assessments of textile fiber harm.^13^ However, many modifications of natural fibers have been shown to decrease fiber biodegradability.^23^^,^^24^^,^^25^ Assessments of natural fiber biodegradability have investigated fiber degradation in simulated environmental scenarios. For example, investigating wool biodegradation in simulated marine conditions, Collie et al.^26^ conclude that wool will not persist in the environment. However, published studies of fiber biodegradation have been found to be unfit for purpose due to their use of environmentally unrepresentative experimental conditions. Moreover, archaeological research has recovered natural fibers, including wool, from fabrics which are centuries or even millennia old recovered from both waterlogged (anoxic) and dry sites including shipwrecks^10^^,^^27^^,^^28^ and caves.^11^^,^^29^ While controlled assessments of fiber biodegradation may reflect general biodegradability trends by fiber types, the archaeological evidence of fiber persistence demonstrates that such assessments cannot be generalized to all natural fibers.

### Consequences of natural fiber persistence

Natural fibers recovered in archaeological studies may pose no contemporary environmental harm themselves. However, their preservation in the environment implies natural fibers, known to dominate present-day environmental samples, can persist for environmentally meaningful time periods. Recognizing this natural fiber persistence, it cannot be assumed that these fibers will not cause environmental harm alongside their plastic analogs. Moreover, the partial degradation of natural fibers (through biodegradation or otherwise) has the potential to accelerate the leaching of any harmful chemicals associated with natural textile fibers into the environment, relative to plastic fibers.^30^ While plastic fibers are known to interact with chemical pollutants,^17^ the properties of natural fibers to act as sorbents for chemical pollutants in pollution remediation efforts has also been identified.^31^^,^^32^ The ecotoxicological impacts of natural fiber ingestion are also largely unknown. Where harm from non-plastic fiber ingestion has been explored, exposure to regenerated cellulose fibers (e.g., lyocell and viscose), and cotton fibers, has been found to elicit ecotoxicological effects in test organisms alongside plastic fibers.^33^^,^^34^^,^^35^^,^^36^

That natural textile fibers persist on timescales from decades to millennia implies that natural textile fibers also have the potential to act as technofossils. Technofossils are microscopic particulates, including microplastics,^37^ spheroidal carbonaceous particles,^38^ and glass microspheres,^39^ associated only with anthropogenic activity and preserved in environmental archives—including aquatic and terrestrial sediments. Lake sediments are a particularly powerful environmental archive for technofossil investigation.^40^ Demonstrating that non-plastic fibers are present in lake sediment records has the potential to extend assessments of historic footprints of human activity beyond currently established technofossil records. Recovery of centuries-old individual fibers is more challenging than studying entire garments or fabric swatches.^41^ But there does exist a body of work detailing the recovery of individual textile fibers from archaeological sites, extending archaeological understanding of material culture histories.^12^^,^^41^

### This study

**Figure 1:**
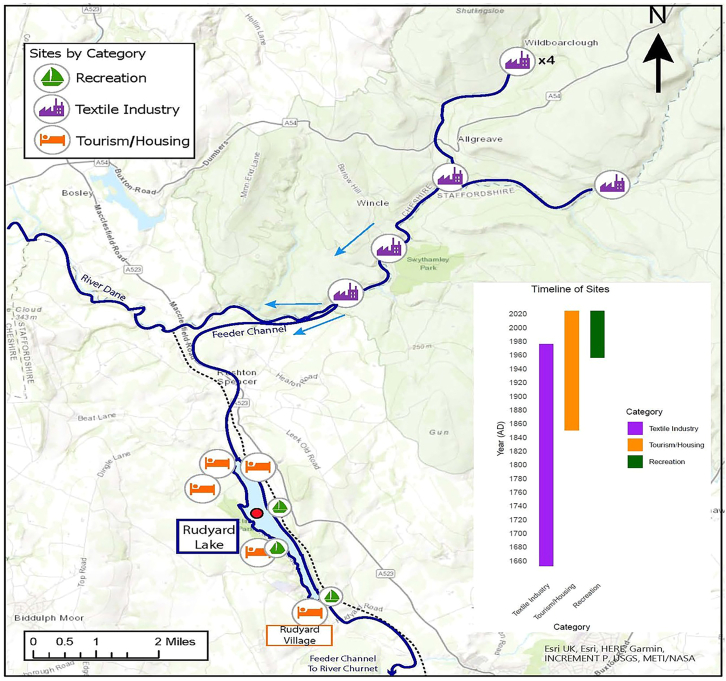
Map of Rudyard lake, Staffordshire, UK. Arrows show flow direction of the river Dane and Dane feeder Icons depict locations of key textile industrial, residential, and recreational activities that are potential textile fiber sources. Black dotted line shows route of railway. Red circle shows approximate coring location. Inset shows timelines of these activities. Screengrabs of OS maps for key sites provided in the supplementary maps (Data S1).

Building on this established archaeological precedent, we present evidence of individual natural textile fiber (cotton and wool) preservation in, and dominance throughout, a ^210^Pb-dated sediment core. This core is recovered from one of the few aquatic depositional environments downstream of a historic center of UK textile manufacture—Rudyard lake, Staffordshire (Figure 1). Records of textile manufacturing upstream of Rudyard lake span approximately 300 years (c.1650s–1970s).

The extrapolated ^210^Pb chronology for this core extends into the late 19th century. Fibers recovered from the bottom of this core were therefore deposited during the UK’s Second Industrial Revolution. Falling between c.1880–1914, this period was characterized by advancements in agriculture, including its commercialization, and industrial science (chemicals, metallurgy, and associated manufacturing industries).^42^ Despite the industrial history of the English West Midlands being well established, there is a paucity of published data related to the Rudyard lake catchment. Site context is derived from oral histories, community social science workshops, and research in local archives. Rudyard lake’s geographical location and industrial heritage, combined with oral histories and associated local expertise, make Rudyard lake a unique site of international importance for textile fiber preservation.

## Results

### Rudyard lake

Constructed from 1797 to 1801 to provide a water source that could ensure navigable water levels on the vital canals of the Trent canal system, Rudyard lake was initially fed by local streams. Rudyard lake flows into the river Churnet, a main tributary of the river Trent. A feeder channel from the river Dane (hereafter called the Dane feeder) was subsequently built (in 1809) to increase the lake’s capacity (Figure 1).

Though the Dane feeder only ran when the river was in spate—6 inches (15.24 cm) above normal weir height—its interference with local water demands was always a source of conflict. The owners of Whitlee’s Mill, immediately upstream of the Dane feeder’s initial connection to the river Dane, had successfully argued that the initial leat (a feeder watercourse for a mill) should be built downstream of the weir that fed their mill to protect their water supply. This initial leat did not supply sufficient water. A parliamentary intervention in 1821 resolved the issue by moving the feeder inlet upstream,^43^ with the leat moved by 1824. The repeal of the Corn Laws (1846) reduced the profitability of mills in this region. As maintenance became more sporadic, flooding along the Dane feeder increased, and this in turn exacerbated disputes over water rights and flood management responsibilities. The feeder channel from the Dane remained open throughout the lifetime of the river Dane mills^43^ that lay upstream of it, and during the period in which they were abandoned and decayed. Maintenance of the Dane feeder finally ended in 2008.

### Rudyard lake’s textile fiber history

Ordnance Survey maps held by the National Library of Scotland^44^ (Data S1) confirm the presence of textile mills (predominantly cotton and silk), color mills, dye houses, and paper mills on maps dating back to the 1870s upstream of the Dane feeder. There were at least six mills at the time of the Dane feeder’s construction^43^ (Figure 1). Paper, at the time, was often made using textile wastes and offcuts.^45^ The uses of these mills changed throughout the 19th and 20th centuries. A color mill is present on the river Dane at Danebridge up to 1947 (Data S1), and the Danebridge Cotton mill was demolished in 1979.

In this project, our community social scientists reviewed archives from textile firms operating in the wider Leek area, including from mills located on the river Churnet. Their work scoped existing histories on the thousands of men, women and children who worked in the Leek region’s textile industry. These archives demonstrated that industrial pollution on the river Churnet, downstream of Rudyard lake, had been a concern for local people since the 1820s. The textile and dye mills were resource-hungry (energy, water, and people) and, though the river Churnet was over-extracted, public concerns about pollution tended to be used as a strategy for productivity gains by mill owners. Where local people brought forward worries about the impact of mill effluent, mill owners argued for the right to abstract more water to dilute their effluent more effectively.

### Fiber sources beyond textile manufacture

Textile mill activities were augmented by the domestic laundry activities of the few households resident in the Rudyard catchment. A commercial laundry operated by the Rudyard Hotel (in Rudyard Village, Figure 1) likely discharged to Rudyard lake around the turn of the 20^th^ century, possibly from 1900 to 1914. A rail line to Rudyard lake was constructed in 1849. From then on, the lake’s picturesque setting and leisure activities attracted up to 20,000 visitors per day. The recreational use of Rudyard lake associated with these visitors and the laundering of textiles using Rudyard lake and the river Dane upstream of it are additional potential historic textile fiber source.

### Rudyard lake sediment core chronology

**Figure 2:**
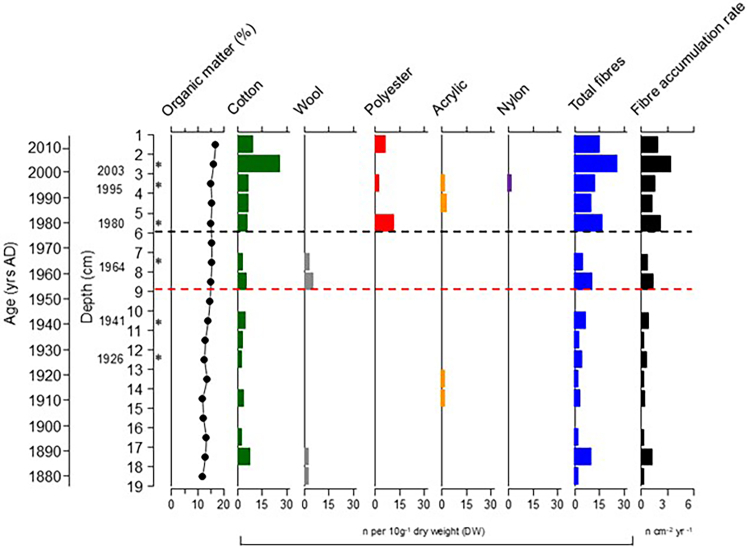
Textile fiber populations throughout the Rudyard lake sediment core, reported as number of fibers per 10 g dry weight (DW) for cotton, wool, polyester, acrylic, and nylon fibers, total fibers and fiber accumulation rate as number of fibers cm^−2^ yr^−1^ from the Rudyard sediment core The black dotted line denotes when accumulation rate changes significantly using change point analysis while the red dotted line denotes the beginning of major use of acrylic in the 1950s. Also displayed is the organic matter as % dry weight and the locations of ^210^Pb dated samples and their associated dates (∗) that fell within the ^210^Pb dating horizon. The 0–1 cm level is excluded from Figure 2 as the water content in this sample was so high it was not possible to recover 10 g (wet weight) of material. The number and color of each recovered textile fiber are provided in Table S3.

Six of the seven dated samples fell within the ^210^Pb dating range (of 100–120 years). The CF:CS dating model was chosen over the CRS model as the excess ^210^Pb inventory in the Rudyard core was significantly less than expected for the UK,^46^ which implies that key assumptions of the CRS model were violated (see SI). This CF:CS age model (Figure S1) constrained a core chronology dating to 1926 at 12.5 cm with a low (and relatively uniform) sedimentation rate of ∼1.3 mm y^−1^ (1σ = 1.1–1.6 mm yr^−1^). This low sedimentation rate is explained by Rudyard lake’s bankside forest and its low and intermittent water supply, detailed in the archival records reviewed above. As the deepest date fell outside of the ^210^Pb dating horizon, the basal age of our core is therefore extrapolated to c.1876 ± 25 years, assuming a continued low and uniform sedimentation rate through the core’s entire length. This is supported by the consistent organic matter profile (Figure 2), sediment lithology and no documentary or historical evidence of major lakeside land use change since this time. Under the CF:CS model we estimate dating errors to be ±10 years at 7.5 cm (1964), ±17 years at 12.5 cm (1926) and ±25 years at the base of the core at 19 cm (∼±1876) (Table S2). These errors are in line with previous microplastic analyses of 210 Pb dated lake sediment cores.^47^^,^^48^ Full details are given in the SI.

### Natural fiber isolation from complex environmental samples (QA/QC)

The number of airborne textile fibers deposited during sample preparation was monitored using an upturned adhesive tape (TapeIt, 3L Office, Denmark). Textile fiber deposition, calculated as fibers deposited per minute, varied from 0.05 to 0.14 fibers per minute during sample preparation, and 0.09 to 0.13 fibers per minute during the transfer of fibers from filter papers to microscope slides (Tables S4 and S5). These tapes were exposed throughout the entirety of these steps, while samples were only exposed during wet sieving and the transfer of fibers from filter papers to slides for identification. The values reported here therefore represent deposition rates over a longer period of time than samples were exposed. The textile fibers deposited on these tapes are detailed in Tables S4 and S5.

### Textile fibers recovered from the Rudyard core

**Figure 3:**
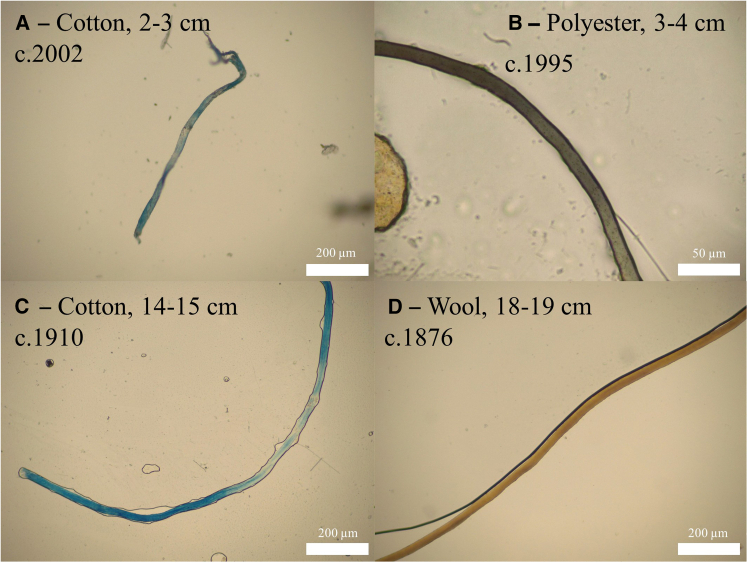
Brightfield micrographs of textile fibers recovered from the Rudyard core Textile fiber type (cotton, A and C; polyester, B; and wool, D) and depth of sediment from which the fibers were recovered and its associated age location indicated in each figure.

Fiber recovery and identification followed standard forensic fiber analysis techniques (see Methods), including the exclusion of all white and colorless fibers. Fibers were isolated using a chemical-free method to ensure fiber integrity was not compromised (see Methods). As this method involved the use of a coarse (350 μm) sieve, in addition to the exclusion of white/colorless fibers, the number of fibers recovered from the Rudyard Lake sediment core is likely an underrepresentation of total fiber abundance. In total, 67 fibers were recovered from the 19 cm sediment record, with fiber recovery decreasing down the core (Figure 2). Textile fibers of five types (cotton, wool, polyester, acrylic, and nylon) were isolated from all but three of the 1 cm sections of the core (15–16 cm, c.1902; 9–10 cm, c.1949; 6–7 cm, c.1972) (Figure 3). Cotton was the most common fiber type accounting for 70% of fibers recovered and was dominant throughout the core’s depth.

Change point analysis identified a shift in total fiber accumulation rates at 5–6 cm depth (c.1979; see STAR Methods). The mean fiber concentration per core section prior to 1979 was 3.68 fibers per 10 g of dry sediment. In this older section of the core, all bar two of the fibers recovered were either cotton or wool, and wool is the only fiber type recovered from the core’s deepest 1 cm section (extrapolated date of c.1876). Acrylic fibers were recovered from two core subsections (c.1920–1930 and c.1930–1940) (*n* = 1 fiber per subsection). These two dates predate the industrial production of acrylic fibers (see Discussion).

After c.1979 (sample depths 1–6 cm), fibers were present in all core subsections and mean fiber concentration increased to 15.88 fibers per 10 g of dry sediment. This change is associated with a particularly marked increase in cotton fibers, and an increase in the diversity of fiber types driven by the occurrence of polyester, nylon, and acrylic fibers (Figure 3). In total, 30 of the core’s 67 fibers (45%) were recovered from this subsection of which 20 were cotton, seven were polyester, two were acrylic, and one was nylon.

## Discussion

### Textile fiber histories in the Rudyard catchment

Extrapolating the age model, the bottom sediment of the Rudyard core is likely to have been deposited in the late 19^th^ century (c.1876 ± 25 years). Recovery of natural textile fibers from these bottom sediments (100% of fibers from c.1876-c.1910, and 90% of fibers from c.1876-c.1972) complements evidence from the archaeological study of fabrics and fibers that demonstrates the persistence of natural textile fibers, as well as plastic fibers, in the environment. The notable increase in textile fibers in the 1970s, identified from change point analysis, may be associated with an introduction of fibers resulting from the demolition of the Danebridge cotton mill in 1976. Values at Rudyard lake are comparable with or higher than fiber concentrations and accumulation rates found in other UK urban settings (e.g., Turner et al.^49^).

Two acrylic textile fibers were found some 20–30 years before their mass production in the UK, one at 12–13 cm (c.1930s ± 17 years) and one at 13–14 cm (c.1920s ± 19 years), though this temporal discrepancy is within or just beyond the error of the dating model. If these dates are considered correct, however, this may be associated with the downward migration of textile fibers through the sediment core Dimante-Deimantovica et al.^48^ suggest that fibrous microplastics exhibit less vertical movement through sediment cores than non-fibrous microplastic particles. An alternative explanation for the presence of these acrylic fibers at these core depths could be bioturbation of deposited sediments, something previously discussed within the context of microplastic recovery from sediments by Bancone et al.^50^ The temporal implications of bioturbation in the Rudyard core could be considerable given its slow sedimentation rate, though no evidence of invertebrate biota or surface or infaunal invertebrate activity was found during core collection or subsampling. We also consider aerial or laboratory contamination during sample processing unlikely as no acrylic textile fibers were identified on the adhesive deposition tapes (Table S6).

Mills upstream of the Dane feeder varied in their activities across their operational lifetime. These mills were part of an 18^th^ and 19^th^ textile industry boom across the wider English Midlands and northwest England. Modeling of the atmospheric transport of microplastic particles has shown that fibrous shapes are likely to be transported greater distances than spherical shapes.^51^ Beyond Rudyard lake’s catchment, nearby activities associated with industrial textile manufacture and trade would have introduced textile fibers into the atmosphere. Airborne textile fibers are a health hazard to textile factory workers to this day,^52^ and the manufacturing process associated with natural textile fiber products have been identified as driving factors in the contemporary dominance of airborne natural fibers over plastic fibers.^53^ Such airborne fiber emission would have been a major pathway for textile fibers at the time that the Churnet catchment mills were operating. Transport of equivalent airborne fibers from industrial activities located beyond Rudyard lake’s catchment cannot be ruled out as an additional source of fibers isolated in this study.

We recognize that the fibers recovered from our sediment core are sequestered and removed from the environment over the long term (i.e., were not environmentally available at the time of core collection). We therefore do not consider these fibers to be a potential source of environmental harm in the present day. However, alongside contemporary assessments of natural fiber dominance in the environment,^1^^,^^2^^,^^3^^,^^4^ complementary archaeological research isolating fabric swatches and individual fibers from different environments,^11^^,^^12^ and recent studies quantifying natural fiber ecotoxicity,^33^^,^^34^^,^^35^^,^^36^ the recovery of non-biodegraded natural textile fibers from the Rudyard lake core suggests that assumptions and assertions of natural textile fiber biodegradability and risk must be revisited.

This study was only resourced to explore this line of inquiry at a single site, and for this reason we do not extrapolate our findings to additional sites within the UK or beyond. This work serves to highlight the ability of natural textile fibers to persist in the sediment record over long time periods. This is a finding that warrants additional investigation at larger spatial scales and under different catchment scenarios. The implications of the work that builds on these findings are of importance to the study of technofossil markers of human activity, and sustainable fashion narratives.

### Natural and non-plastic fibers as technofossils

Contemporary assessments of textile fiber pollution in aquatic environments have associated elevated textile fiber concentrations with proximity to sources of textile manufacture.^54^ Textile fibers have also dominated UK lake sediment records of microplastic pollution since the 1950s.^49^ However, depositional aquatic environments downstream of historic centers of textile production, where the water-powered mechanized industrial production of textiles emerged in the 18^th^ century, are rare. This enables our results from Rudyard lake to consider an internationally unique framing of natural fibers as a potential technofossil marker of human activity.

The findings presented here, to the authors’ knowledge, are the first study to explore the preservation of natural textile fibers in aquatic sediments in the contexts of environmental pollution or sustainable fashion. However, previous environmental pollution assessments of textile fibers in recent (late 20^th^ century) aquatic sediments have isolated non-plastic textile fibers from environmental archives.

This work includes the recovery of regenerated cellulosic fibers (e.g., rayon and viscose from lagoon sediments in Mexico dating to the late 1950s).^47^ Likewise, Brandon et al^55^ also quantify fibers as the dominant microplastic morphology in Californian coastal oceanic sediments recovered from core sections that pre-date plastic’s mid-twentieth century mass production. Consideration of natural fibers is beyond the scope of Brandon et al^55^ and, because this was not their focus, they understandably did not undergo the necessary QA/QC procedures to consider natural fibers in their cores. However, close inspection of the imagery in their publication (see Figure 2A in Brandon et al^55^) indicates the presence of fibers identified as plastic but which resemble the morphology of cotton (regularly described as a flat, twisted ribbon) (Figure 3). Brandon et al.^55^ suggest that the presence of natural fibers in their samples is likely due to contamination, which cannot be ruled out given their research focus and associated appropriate experimental procedure. However, the recovery of cotton and wool fibers from the Rudyard lake core indicate that contamination may not be the only source of natural fibers in previously published research that details technofossils in pre-1950 aquatic sediments.

Further research is required focusing on other centers of historic textile production and in locations where histories of textile manufacture and/or care (e.g., surface water laundry) are known and closely associated with aquatic environments. Such work requires careful consideration of the methods it uses to isolate fibers, given the potential fragility of the fibers that might be encountered. The no-chemical method employed in this study was chosen to maximize fiber integrity and recovery. We recognize that our use of a coarse (350 μm) sieve has likely resulted in an underrepresentation of fibers and fiber concentrations. However, additional non-intrusive methods of particle identification have the potential to overcome the limitations of both physical and chemical microplastic quantification. X-ray CT-scanning of soil cores has been shown to identify microplastic particles without the need for core sub-sectioning or soil handling.^56^ Though Trusler et al.^56^ acknowledge that the dimensions of fibers may challenge the application of this approach to quantify textile fibers in sediment cores, refinement of this method could have the potential to minimize sample contamination in palaeo textile fiber research. If suitable for natural fibers as well as plastic fibers, X-ray CT-scanning would also reduce the risk of aged textile fiber damage or loss associated with mechanical and chemical methods of sediment processing, and would also provide a means to critically test fiber extraction methods and associated QA/QC protocols on CT-scanned cores.

### Implications for the anthropocene

Although the Anthropocene’s definition as a geological epoch has been rejected by the International Commission on Stratigraphy,^57^ it is worth considering the inclusion of natural fibers as technofossils in recognition of the enduring impact of the Anthropocene concept on environmental research, culture, and society. Technofossil records in environmental archives, primarily from the 1950s to the present day, were used to support a geological definition of the Anthropocene—a concept that describes the current geological epoch as one characterized by human activity that has fundamentally changed the Earth system and its natural processes.^58^ It was because of their persistence and ubiquity in the environment that microplastic fibers and particles were identified as a key technofossil type in this debate.^59^ Microplastic fibers were identified as more suitable than microplastic particles in efforts to define the Anthropocene due to their more limited movement within sediment cores.^48^ Our findings show that natural fibers are also preserved as technofossils in the sediment record, highlighting the enduring and variable legacy of material cultures and resource exploitation in environmental records. The importance of this within sustainability debates persists within the cultural framing of the Anthropocene, irrespective of its geological status.

Indeed, natural textile fibers, whose use long pre-dates that of plastic fibers, was not formally considered within discussions of the geological Anthropocene. However, as the social concept of the Anthropocene evolves, it is evident that textile fibers of all types represent perhaps the most tangible technofossil connection between society’s contemporary material cultures and enduring environmental footprints. Future natural fiber technofossil research, extending beyond the geological Anthropocene’s mid-twentieth century focus, should be central to efforts to explore both the range of technofossils that record humanity’s accelerating planetary impact, and the cultural Anthropocene—a concept rich with opportunity in future sustainability debates and action.^60^

### Natural fibers and sustainable material narratives

Accurate assessments of textile fiber pollution necessitate thoughtful integration of complementary disciplines.^21^ The environmental impacts of natural textile fibers were first identified a decade ago.^30^ Since then, the environmental pollution research community has made no meaningful progress toward establishing a holistic understanding of textile fiber impacts on the environment. Research instead remains focused on the (petrochemical) polymer type, rather than the (fiber) product.^61^ We therefore call for an urgent response from environmental pollution researchers to incorporate natural textile fibers into research that attempts to establish the relative environmental harms associated with the representative total population of textile fibers—of which natural textile fibers constitute the bulk. If evidence of harm is found, this body of work must inform updated recommendations for material use and marketing within the fashion and textiles industry to avoid wasteful and regrettable material substitutions that have occurred with other plastic alternatives.^62^

Societies worldwide must reduce their consumption of plastic and mismanagement of plastic waste. Appropriate material substitutions are one way this can be achieved. However, here we find that “natural” textile fibers—specifically cotton and wool—persist in the sediment record for c.150 years, and in greater abundance than their plastic analogs. These results highlight the persistence of individual natural fibers as both particulate pollutants and technofossils. While the prevalence and persistence of plastic textile fibers in the environment is rightly a cause for concern, it is potentially dangerous to exclude natural fibers from fiber pollution research. However, while there exists an extensive body of research documenting the ubiquity and environmental harm associated with plastic fibers, this work has led to the untested promotion of natural textile fibers as “greener” material substitutions. This is, at least in part, based on poorly tested assumptions of biodegradability. Considering our findings in the context of contemporary assessments of environmental textile fiber populations, we conclude that statements of natural fiber biodegradability may be misleading.

Natural textile fibers have considerable potential to extend technofossil assessments of human impacts on the environment, and in turn contribute to the cultural concept of the Anthropocene. We therefore call for an urgent response from researchers to thoroughly assess the environmental prevalence, persistence, and impacts of all textile fiber types. This is necessary to ensure “green” claims about material choices within the fashion and textiles industry and associated policy are appropriately informed.

### Limitations of the study

The data presented—and the chemical-free method of sediment processing used to recover them—represent an important first step in the assessment of textile fiber persistence in sedimentary environmental archives. However, this study recovered textile fibers from only one sediment core. This limits our ability to consider the spatial extent of textile fiber preservation in similar environments. The sediment record we recovered also only spanned 150 years. When considering natural textile fibers as technofossils, similar assessments must be considered using environmental archives that predate this sedimentary archive.

## Resource availability

### Lead contact

Requests for further information and resources should be directed to and will be fulfilled by the lead contact, Thomas Stanton (t.stanton@lboro.ac.uk).

### Materials availability

This study did not generate new unique reagents.

### Data and code availability

• Data: All data relating to the Rudyard core and fibers recovered from it are available in the main text or the Supplementary Materials. A public report summarizing community input into social science and background data generation for this project is available online via Fashion Revolution at https://www.fashionrevolution.org/restorying-riverscapes/. Workshop notes are confidential under protection of personal privacy.
• Code: This study generated no custom code.
• Other: For any other requests, please direct correspondence to the lead contact

## Acknowledgments

This work would not have been possible without the participation of members of the public who engaged with this work’s Churnet Valley textile pollution and textile history workshops as part of the Restorying Riverscapes Project (https://www.fashionrevolution.org/restorying-riverscapes/). This includes Margaret Clark, Dave Bellfield, and Cathryn Walton. We have synthesized their workshop contributions with details published in historical sources that they suggested to us. We also extend our thanks to the staff of Rudyard lake, especially Ray Perry, for hosting us on multiple site visits, facilitating core collection through use of their boat, and always extending a friendly welcome.

We also thank the laboratory staff of 10.13039/100010052Northumbria University Department of Applied Science and Loughborough University Department of Geography and Environment for supporting sample processing and analysis. This work was supported by the following funding sources: c*NERC Discipline Hopping for Discovery Science 2022* award (grant reference: NE/X018091/1) (T.S., K.S., and C.K.-O.). A trilateral fund from the 10.13039/501100000270Natural Environment Research Council, 10.13039/501100000267Arts and Humanities Research Council, and 10.13039/501100006041Innovate UK, through the 10.13039/100014013UKRI Circular Fashion and Textiles Programme: NetworkPlus (grant NE/Y004035/1) (T.S., K.S., and C.K.-O.); The 10.13039/501100001961AXA Research Fund Fellowship (T.S.); 10.13039/501100000857Loughborough Universities Vice Chancellor Independent Research Fellowship (T.S.) are also acknowledged. Pilot funding provided by Keele University’s Keele Active Partnership Programme via Fashion Revolution for “Restorying Riverscapes” comprising three community workshops (D.M., T.S., A.L., C.S., and A.Wheat.). 10.13039/501100005044Keele University’s Policy Engagement Fund for “Unravelling Fibreshed” for a workshop on textile fibers and pollution policy development (D.M., A.L., and T.S.). Keele University Faculty of Natural Sciences pump-priming funds to hold two follow-on community validation workshops post-report (D.M. and A.L.).

## Author contributions

Conceptualization, T.S., A.L., C.S., and D.M.; methodology, T.S., A.L., C.S., D.M., S.W., K.J.S., C.K.-O., A. Wheat, A. Wood, A.B., N.R., P.G., and A.C.; investigation, T.S., A.L., C.S., D.M., S.W., K.J.S., C.K.-O., A. Wheat, A. Wood, A.B., N.R., P.G., and A.C.; visualization, T.S., S.W., and A.L.; supervision, D.B.R. and E.B.; writing – original draft, T.S. and D.M.; writing – review and editing, T.S., A.L., C.S., D.M., S.W., K.J.S., C.K.-O., A. Wheat, A. Wood, A.B., N.R., D.B.R., E.B., P.G., and A.C.

## Declaration of interests

K.J.S. and A.B. are employed by The Microfibre Consortium, though all work associated with this manuscript was undertaken in their capacities as university researchers and independent scholars, respectively. All other authors declare they have no competing interests.

## STAR★Methods

### Key resources table

**Table:** 

REAGENT or RESOURCE	SOURCE	IDENTIFIER
**Deposited data**		

Raw and analyzed data	This paper	doi
Historic maps	National Library of Scotland	https://maps.nls.uk/geo/find/marker/

**Software and algorithms**		

R 4.4.2	R Core Team	https://cran.r-project.org/
Changepoint 2.3	R Package	https://cran.r-project.org/web/packages/changepoint/index.html

### Method details

#### Archival methods, site selection, and heritage

Lying approximately 4 km northwest of the industrial-era mill town of Leek, Rudyard Lake was identified as a suitable site for palaeo-limnological textile fiber analysis following extensive evaluation of archived UK Ordnance Survey Maps spanning the English Midlands, North of England, and Scotland (Data S1). Ordnance Survey Maps show very few depositional lentic environments downstream of the UK’s historic textile industry centers. We are not able to say with absolute certainty that all the fibers recovered from the Rudyard sediment core originated from the mills identified upstream of Rudyard Lake (Figure 1). In addition to the local activities associated with Rudyard Lake (Figure 1), it is also possible that nearby centers of textile manufacturing during the period our core covers (e.g., Nottinghamshire, Derbyshire, Manchester) could have been a source of fibers isolated from this core, transported by air to Rudyard Lake. However, the mills upstream are the most proximate, and therefore likely, source.

To extend our understanding of the Rudyard catchment, working with a team of citizen social scientists, we reviewed archives from textile businesses, scoped existing histories on the thousands of Leek men, women and children who worked in the industry, as well as the contribution of Huguenot refugees to the training and employment of local Leek workers. With assistance from the Nicholson Institute in Leek and local historians Cathryn Walton and David Belfield, we assembled resources on industrial heritage. We also compiled narratives of the post-industrial river and lake recovery in the area by collecting oral and photographic histories of the riverscape to map the historic locations of mills and dyeworks and bring to life the area’s textile heritage.

#### Sediment collection and analysis

A 19 cm sediment core from the northern end of Rudyard Lake (mean depth 4.4m) (Figure 1) was recovered using a Kajak gravity corer on 22/03/2022^63^ with a polycarbonate sampling tube of 86 mm internal diameter. Prior to core collection, the tube was submerged under the surface of the lake and agitated to remove any potential contamination fibers from its internal walls prior to core collection. Prior to coring the construction and dredging history of Rudyard Lake was investigated to identify the most suitable site for coring (Figure 1). Due to the accumulation of sediment at the dam head (located at the southern end of the lake in Rudyard village), dredging did take place in the 1990s. However, records confirm that dredging only took place close to the dam head. Consequently, we identified a coring site at the northern end of the lake to ensure a continuous sediment record.

The core was extruded in the field into 1 cm sections which were then transferred into labeled transparent LDPE sample bags and stored in a refrigerator at 4°C until analysis. Organic matter (OM) content was measured for contiguous 1 cm samples following the loss on ignition method (LOI).^64^ Wet bulk density was determined for each 1 cm sample by weighing a 2 cm^3^ sample of sediment, subsequently dried at 125°C for 12 h to measure the water content and dry bulk density.

#### Sediment dating

Dried sediment samples (Table S1) were radiometrically dated through ^210^Pb dating, via a proxy method using measurement of its granddaughter radionuclide ^210^Po via alpha spectrometry (Figure S1, Table S2). The method is based on that outlined in Flynn,^65^ whereby an aliquot of the sample was spiked with ^209^Po as a chemical yield monitor and digested with aqua regia solution. The Po was then separated by auto-deposition onto a freshly cleaned silver disc and counted by alpha spectrometry to determine the ^210^Po activity. The limit of detection was 0.1 Bq kg^−1^. Sediment accumulation rates were calculated using the Constant Flux: Constant Sedimentation CF: CS model^66^ of ^210^Pb dating, by plotting the natural logarithm of the unsupported ^210^Pb activity (^210^Pb_excess_) against depth, and using the Constant Rate of Supply (CRS) model of ^210^Pb dating.^67^ Supported ^210^Pb activities (ca. 0.055 Bq g^−1^) were estimated using average activities at the core base, i.e., the activity at which ^210^Pb declines to near-constant values at depth in older core material,^68^ and confirmed through gamma spectrometry (using HPGe detectors) via measurement of the ^210^Pb daughter product ^214^Pb.

#### Sediment processing

The isolation of natural textile fibers from lake sediments cannot rely on standard palaeo-ecological methods of sediment processing, or on standard analytical methods used for microplastic identification. Palaeo-ecological methods typically use chemicals that have the potential to damage natural fibers, particularly those that may have been buried in lake sediments for decades, or even centuries, such as concentrated (30%) hydrogen peroxide.

Fibers were isolated from wet sediments to ensure sample handling and drying did not compromise the integrity of aged fibers. The mass of wet sediment from which fibers were isolated was either ∼20 g for wetter, looser sediments at the top of the core (1–4 cm), or ∼10 g for all other samples. Each subsample was transferred into a clean 100 mL glass beaker that had been triple rinsed with deionised water. This material was washed through a 350 μm sieve using gentle agitation and deionised water from a wash bottle. Preliminary experimentation found finer mesh apertures (63 μm and 125 μm) to be unsuitable for this methodology, as they required longer processing times (increasing potential for contamination) and retained a greater volume of fine sedimentary material that hindered textile fiber recovery.

Once the water passing through the 350 μm sieve ran clear to the eye, the retained sample was transferred back into the glass beaker using more deionised water before being vacuum filtered through a Whatman Grade 1 qualitative circle filter paper. Once filtered, the filter paper was transferred into a plastic Petri dish and sealed with the lid on using electrical tape until the sample was analyzed. Whenever the glass beaker was not being used, it remained covered to minimise aerial contamination.

#### Textile fiber recovery

Filter papers were first examined using stereomicroscopy (x10-40, Leica S6E, Germany). The filters were systematically inspected under low powered microscopy, following horizontal transects to ensure comprehensive coverage of the entire filter paper. Subsequently, any colored fibers identified were carefully retrieved using stainless-steel tweezers and individually mounted on glass slides (CIMED, 1–1.2 mm thick, 25 × 75 mm) using glycerol mountant (VWR CAS number: 56-81-5). Each recovered fiber was covered with a round coverslip (Ø 9 mm, Thermo Scientific, Germany). The entire procedure was carried out under the low power microscope to minimise the potential for contamination as per standard forensic fiber examination protocols.^69^

#### Textile fiber identification and quantification

Glass slides containing mounted fibers were examined using Polarising Light Microscopy (PLM) (Leica DM2700P, Germany) with magnification of x100-400. This is a standard forensic science textile fiber identification technique.^69^ PLM overcomes the challenges of established spectrographic microplastic identification techniques used for polymer identification (e.g., FTIR or Raman spectroscopy) when analysing naturally occurring polymers that can be anthropogenically modified for textile fiber applications, but which retain similar chemical signatures to their natural polymer origins (e.g., cellulose and keratin).^13^ Natural fibers were identified without polarising light filters based on their morphological characteristics, such as the presence of scales for woollen fibers, or flat ribbon-like twists in cotton fibers, while synthetic fibers were identified based on their interaction with polarised light using the polarising light filters.^2^ Concentrations of fibers were calculated as numbers of fibers per dry mass of sediment analyzed (particles per 100 g). Net fiber accumulation rates (no. cm^−2^ yr^−1^) were calculated by multiplying fiber concentration by the ^210^Pb-derived sediment accumulation rate. Fiber concentration for each depth/age interval assumes uniform distribution of microplastics in each whole core slice.

#### QA/QC

The fiber recovery process was conducted with strict adherence to laboratory protocols to minimize the risk of sample contamination arising from airborne fiber deposition. Procedural blanks were processed using two repeats of 500 mL of distilled water, from which just one cotton textile fiber was recovered. Colourless fibers were not examined in line with forensic science fiber identification best practice.^69^ Therefore, clean, white cotton laboratory suits made from colourless cotton fibers were worn, along with nitrile gloves, during sample processing. Prior to commencing the fiber recovery, the work bench was wiped clean. Subsequently, upturned adhesive tape (TapeIt, 3L Office, Denmark) was strategically positioned on the workbench to monitor any airborne deposition that might occur during the work. Upon completion of the microscopy, the adhesive tape was carefully affixed to a transparent acetate sheet and examined for the presence of colored fibers. The number of fibers deposited, and the duration of the tape’s exposure was noted. Fiber deposition per minute was estimated using these values (Tables S1 and S2).

### Quantification and statistical analysis

Change point analysis was conducted in R to identify shifts in the mean and variance of total fiber accumulation rate over time. The Pruned Exact Linear Time (PELT) method was applied from the “changepoint” package,^70^ with a minimum Bayesian Information Criterion (MBIC) penalty to prevent overfitting and ensure reliable detection of meaningful shifts, even with a smaller dataset. The detected change points were mapped to corresponding years. While a larger sample size is generally preferred for statistical robustness, use of change point analysis here is useful for this unique core with inherent resolution constraints to provide coarse-scale evaluation of shifts.
