# Supplementary material for: “Natural” fibers in lakes: A 150-year sedimentary perspective on persistence
Source: iScience. 2026 Feb 3;29(3):114904. doi: 10.1016/j.isci.2026.114904 (PMC12955082; doi:10.1016/j.isci.2026.114904)
Supplement: Document S1. Figure S1, Tables S1–S5, and Data S1 [file mmc1.pdf]

iScience, Volume *29*

Supplemental information

“Natural” fibers in lakes: A 150-year sedimentary perspective on persistence

Thomas Stanton, Antonia Law, Carry Somers, Savannah Worne, Kelly J. Sheridan, Chimdia Kechi-Okafor, Alana Wheat, Alexander Wood, Anna Bateman, Naomi Richardson, Edwin Baynes, David B. Ryves, Pawel Gaca, Andrew Cundy, and Deirdre McKay

Figure S1. Rudyard core ^{210}Pb activities, and dating models applied. Error bars are shown as solid horizontal lines. Related to Figure 2.

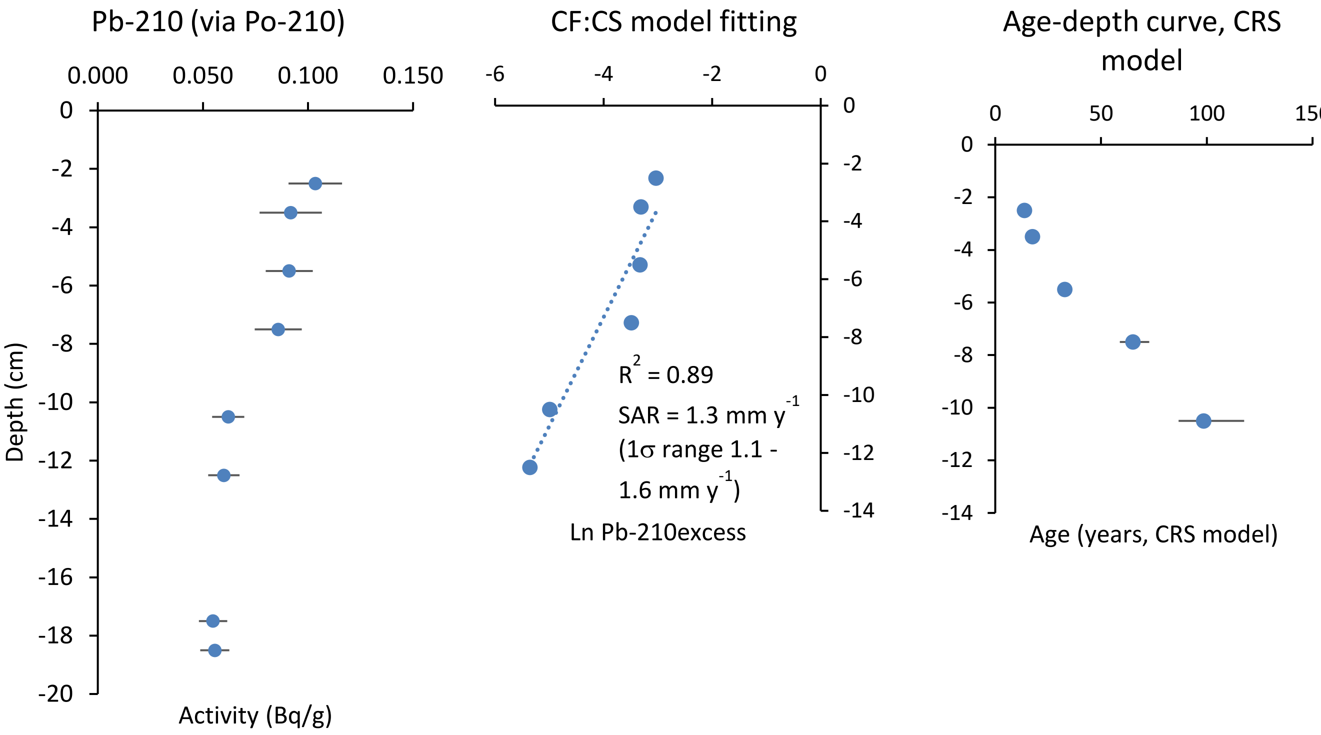

Table S1: Pb-210 (via its granddaughter radionuclide Po-210) specific activities in Rudyard Core. Supported activity estimated using value of constant Po-210 activity at depth, confirmed via determination of the ^{210}Pb daughter product ^{214}Pb (assuming secular equilibrium). Supported Pb-210 = 0.055. Related to Figure 2.

		**Polonium-210**	
**Depth (cm)**	**Dry Mass (g cm^{-3})**	**Bq g^{-1}**	**±**
-2.5	0.133	0.103	0.013
-3.5	0.191	0.092	0.015
-5.5	0.254	0.091	0.011
-7.5	0.281	0.086	0.011
-10.5	0.312	0.062	0.008
-12.5	0.348	0.060	0.007
-17.5	0.326	0.055	0.007
-18.5	0.344	0.056	0.007

Table S2. Calculated age for each dated sample and bottom sample (18.5 cm) from the Rudyard Lake core, including possible date range and error for each sample. Related to Figure 2.

**Age-depth - simple model**			**Lower**	**Upper**	**Error +**	**Error -**
**Depth (cm)**	**Elapsed time**	**Age**	**Date**	**Date**	**yr**	**yr**
2.5	19.20	2003	2000	2006	3.04	3.54
3.5	26.88	1995	1991	2000	4.36	4.86
5.5	42.23	1980	1973	1987	6.99	7.49
7.5	57.59	1964	1955	1975	9.62	10.12
10.5	80.63	1941	1928	1955	13.57	14.07
12.5	95.99	1926	1910	1943	16.20	16.70
18.5	142.06	1880	1856	1905	24.09	24.59

Table S3. Number and colour of each textile fibre recovered from the Rudyard Core. Related to Figure 2 and Figure 3.

Depth (cm)	Fibre type	Colour	Count
0-1	Acrylic	Blue	1
0-1	Nylon	Black/Grey	1
0-1	Cotton	Black/Grey	5
0-1	Cotton	Black/Green	5
0-1	Cotton	Blue	4
1-2	Polyester	Black/Grey	2
1-2	Cotton	Black/Green	2
1-2	Cotton	Green	1
2-3	Cotton	Black/Grey	1
2-3	Cotton	Black/Green	7
2-3	Cotton	Blue	1
2-3	Cotton	Green	1
3-4	Acrylic	Black/Grey	1
3-4	Nylon	Purple	1
3-4	Polyester	Black/Grey	1
3-4	Cotton	Black/Grey	1
3-4	Cotton	Black/Green	1
3-4	Cotton	Pink	1
4-5	Acrylic	Blue	1
4-5	Cotton	Blue	2
5-6	Polyester	Black/Grey	2
5-6	Polyester	Black/Green	1
5-6	Polyester	Green	1
5-6	Cotton	Black/Green	1
5-6	Cotton	Blue	1
7-8	Cotton	Pink/Red	1
7-8	Wool	Blue	1
8-9	Cotton	Blue	2
8-9	Wool	Green	1
8-9	Wool	Black/Grey	1
10-11	Synthetic	Black/Grey	1
10-11	Cotton	Black/Green	1
10-11	Cotton	Purple	1
11-12	Cotton	Black/Grey	1
12-13	Acrylic	Blue	1
12-13	Cotton	Blue	1
13-14	Acrylic	Black/Grey	1
14-15	Cotton	Blue	2
16-17	Cotton	Blue	1
17-18	Cotton	Black/Grey	1
17-18	Cotton	Blue	1
17-18	Cotton	Yellow	1
17-18	Wool	Yellow	1
18-19	Wool	Brown	1

Table S4: Number of fibres recovered from adhesive tapes during wet sieving and filtering of samples. Related to Figure 2.

Adhesive tape number	Number of Fibres from Atmospheric Deposition	Duration (Mins)	Fibre deposition / minute
1	10	120	0.08
2	8	95	0.08
3	11	203	0.05
4	16	117	0.14

Table S5: Number of fibres recovered from adhesive tapes during the transfer of textile fibres from filter papers to microscope slides for fibre identification. Tape 3 includes the processing of three samples that were not taken from the Rudyard core, but were processed in the same batch as Rudyard core sample 18-19cm. Related to Figure 2.

<table>
<tr><th>Adhesive tape number</th><th>Samples Examined</th><th>Number of Fibres Recovered from Samples</th><th>Number of Fibres from Atmospheric Deposition</th><th>Duration (Mins)</th><th>Fibre deposition / minute</th></tr>
<tr><td rowspan="2">1</td><td>0-1cm</td><td rowspan="2">27</td><td rowspan="2">13</td><td rowspan="2">120</td><td rowspan="2">0.11</td></tr>
<tr><td>1-2cm</td></tr>
<tr><td rowspan="7">2</td><td>2-3cm</td><td rowspan="7">48</td><td rowspan="7">23</td><td rowspan="7">270</td><td rowspan="7">0.09</td></tr>
<tr><td>3-4cm</td></tr>
<tr><td>5-6cm</td></tr>
<tr><td>10-11cm</td></tr>
<tr><td>11-12cm</td></tr>
<tr><td>14-15cm</td></tr>
<tr><td>17-18cm</td></tr>
<tr><td rowspan="4">3</td><td>18-19cm</td><td rowspan="4">18</td><td rowspan="4">23</td><td rowspan="4">180</td><td rowspan="4">0.13</td></tr>
<tr><td>Non-sediment sample 1</td></tr>
<tr><td>Non-sediment sample 2</td></tr>
<tr><td>Non-sediment sample 3</td></tr>
</table>

Table S4: Colour, ID, and notes for each textile fibre recovered from upturned deposition tapes. Related to Figure 2.

Recovered Number	Colour	SD/P/L/N/A *	ID
1	Green	P	Regen Cellulose
2	Blue	SD	Polyester
3	Red	N/A	Cotton
4	Brown	P	Regen Cellulose
5	Dark Grey	P	Regen Cellulose
6	Grey	N/A	Cotton
7	Blue/Grey	SD	Polyester
8	Blue	L	Regen Cellulose
9	Grey	N/A	Wool
10	Light Grey	N/A	Wool
11	Dark Blue	L	Polyester
12	Grey	N/A	Cotton
13	Brown	P	Regen Cellulose
14	Grey	SD	Polyester
15	Grey	L	Regen Cellulose
16	Grey	L	Regen Cellulose
17	Dark Blue	L	Regen Cellulose
18	Dark Grey	N/A	Cotton
19	Green	SD	Polyester
20	Grey	SD	Polyester
21	Blue	P	Regen Cellulose
22	Green	P	Regen Cellulose
23	Dark Grey	L	Regen Cellulose
24	Blue	P	Regen Cellulose
25	Dark Blue	L	Regen Cellulose
26	Blue	SD	Polyester
27	Red	N/A	Cotton
28	Brown	P	Polyester
29	Blue	L	Regen Cellulose
30	Light Blue	SD	Polyester
31	C.Less	P	Regen Cellulose
32	Grey	N/A	Cotton
33	Grey	L	Regen Cellulose
34	Grey	N/A	Cotton

(Fibres 21 and 24 fibres turned orange under X polars and blue when parallel on sensitive tint. ID'd as regenerated cellulose and orange colour under X polars was due to the dye on the fibres).

(*) SD = Semi Dull, P = Pigmented, L = Lustrous and N/A = Not Applicable.

Data S1: Supplementary Maps showing the textile industry sites located upstream of Rudyard Lake. Related to Figure 1.

Cheshire Sheet XLIV – published 1881(*44*)

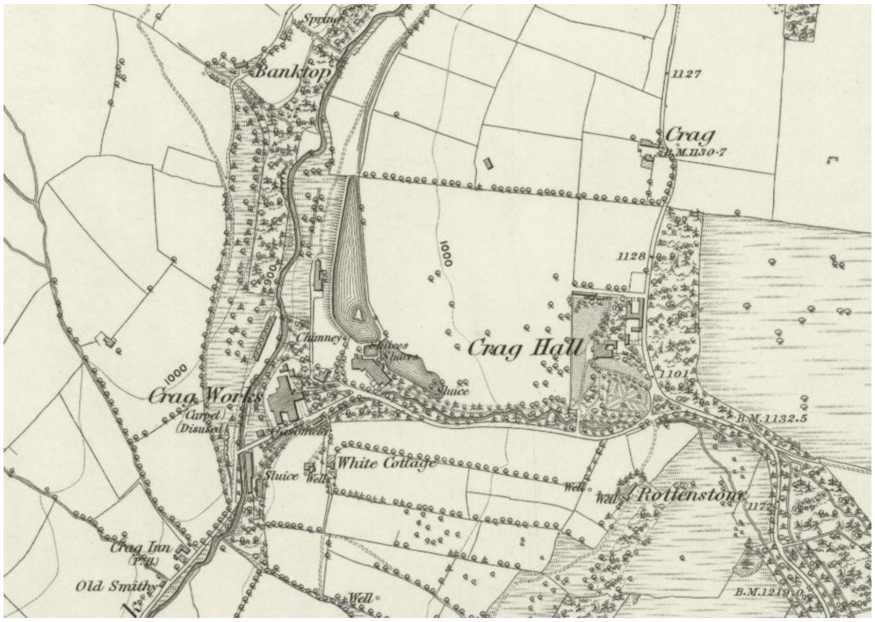

The Crag works site, Wildboarclough

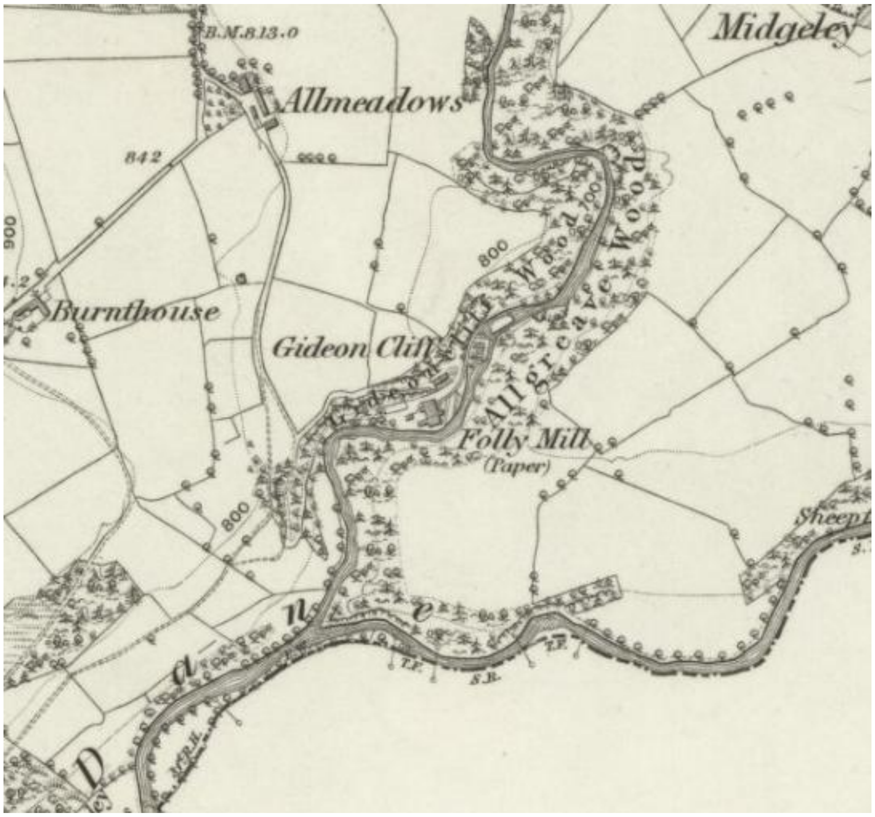

Folly Paper mill, Algreave

Staffordshire Sheet IV.NW – published 1888 (*68*)

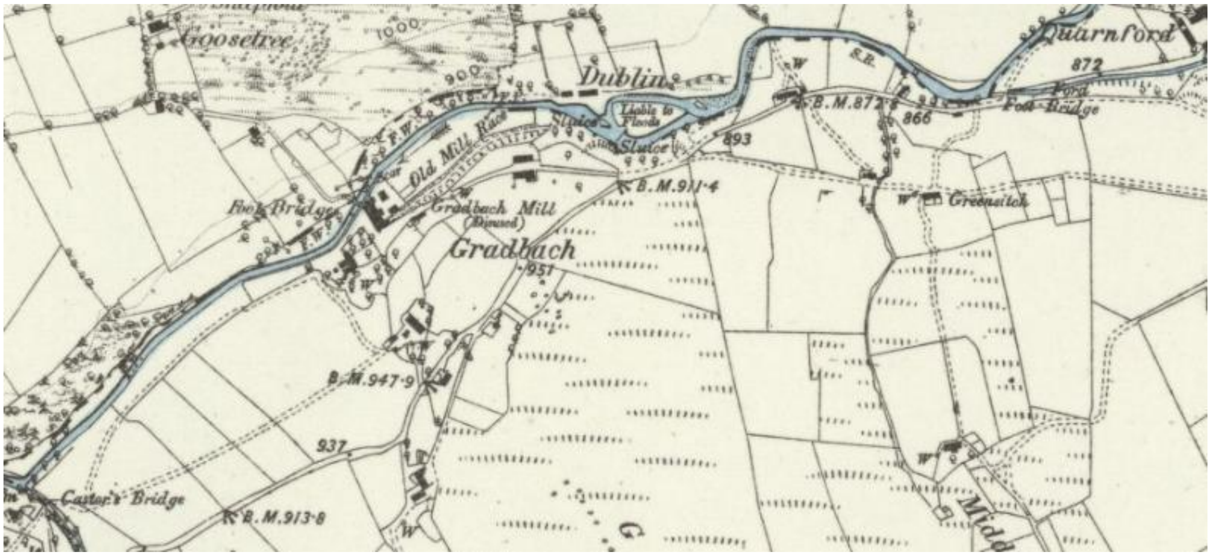

Gradbach mill, Gradbach

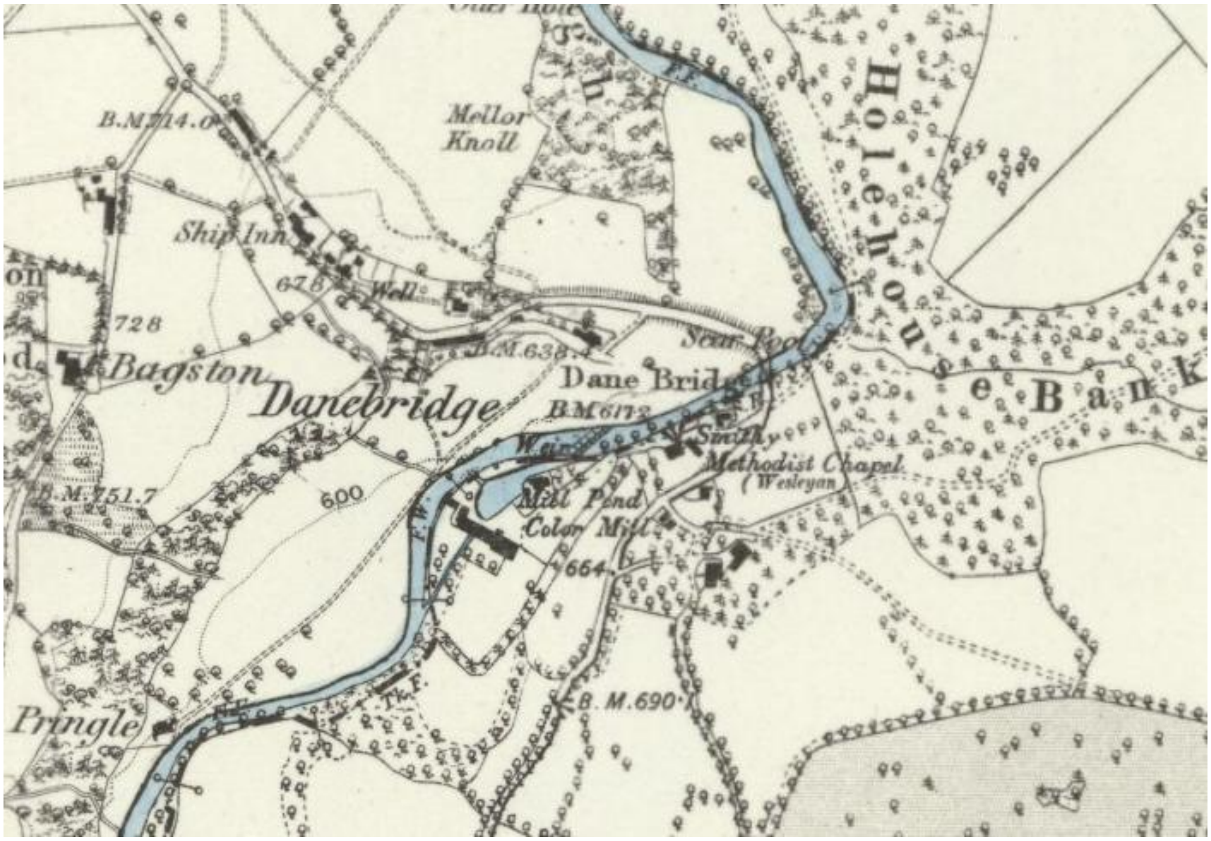

Color Mill, Danebridge

Staffordshire Sheet III.NE – published 1888 (*69*)

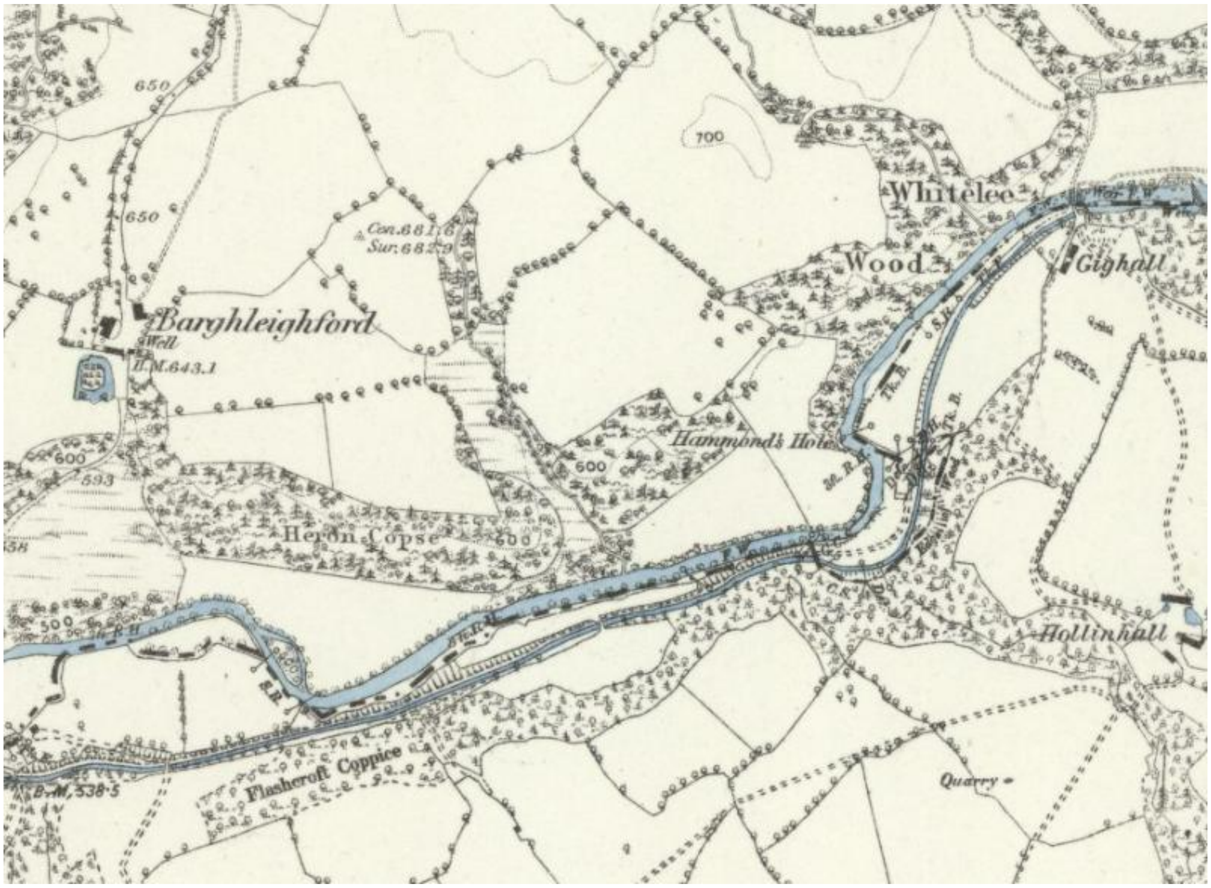

Rudyard feeder channel diverting from the River Dane
